# Is the judicialization of health care bad for equity? A scoping review

**DOI:** 10.1186/s12939-019-0961-y

**Published:** 2019-06-03

**Authors:** Tatiana S. Andia, Everaldo Lamprea

**Affiliations:** 1 Facultad de Ciencias Sociales, Universidad de los Andes, Carrera 1 # 18A-12 Bogotá, Colombia; 2 Asociación Colombiana de Ciudades Capitales , Carrera 9 # 80-45 Oficina 901 Bogotá, Colombia

**Keywords:** Right to health, Judicialization, Equity, Latin America

## Abstract

**Background:**

The term “judicialization of health care” describes the use of rights-based litigation to demand access to pharmaceuticals and medical treatments. The judicialization of health care in Latin America has two defining features. Firstly, it has been conducted in an *individualized* fashion. Secondly, it is highly *pharmaceuticalized*, since most public expenditure related to health rights litigation is invested in paying for costly medications. Recent studies also suggest that the judicialization of health care is bad for equity since it skews limited health resources away from the poorest citizens and in favor of the more affluent.

**Methods:**

We used a scoping methodology to analyze the study-design and the quality of the data employed by the literature that explicitly assesses the impact of the judicialization of health care on equity in Latin American countries. Articles were selected on the basis of their use of an empirical strategy to determine the effect of the judicialization on equity. We searched Google Scholar, PubMed, Scopus, and Scielo databases. We also went through the studies’ bibliographic references, and hand-searched key journals and authors.

**Results:**

Half of the studies analyzed find that judicialization has a negative impact on equity, but the other half finds that evidence is inconclusive or that the judicialization of healthcare has a positive effect on equity. The majority of the studies that collect their own data rely on limited samples that are sometimes not representative and mostly not generalizable. Only few studies conduct systematic comparative analysis of different cross-country or within-country cases. None of the studies reviewed aim to establish causation between judicialization and health outcomes.

**Conclusions:**

We conclude that in order to prove or disprove that the judicialization of health care is at odds with equity we first need to overcome the methodological and research-design problems that have beleaguered the available empirical studies. We also conclude that pharmaceuticals’ price regulation, state capacity, the behavior of litigants, prescribers and judges, and the economic interests of big-pharma, are variables that have to be incorporated into a rigorous empirical literature capable of assessing the regressive effect of health rights’ litigation.

**Electronic supplementary material:**

The online version of this article (10.1186/s12939-019-0961-y) contains supplementary material, which is available to authorized users.

## Background

Most studies on the judicialization of health care focus on Latin American countries like Colombia, Brazil, Costa Rica and Argentina, where litigation of health rights has sharply increased since the early 2000s [1]. According to the available data, in no other region of the world is the judicialization of health care more prevalent than in Latin America. For example, a comparative study found that the number of yearly health rights lawsuits per one million individuals was 3289 in Colombia, 206 in Brazil, 109 in Costa Rica, 29 in Argentina, and only 0.3 in South Africa and 0.2 in India [2].

The term “judicialization of health care” describes the use of rights-based litigation to demand access to pharmaceuticals and medical treatments. Medical malpractice lawsuits, a common subject in health law studies [3], falls within the purview of professional negligence and is usually excluded from the judicialization of health care literature. According to the extant literature, the judicialization of health care in the region has two defining features that set it apart from other comparable cases. Firstly, in highly litigious Latin American countries the judicialization of health care has been conducted in an *individualized* fashion by thousands of plaintiffs who, acting separately, routinely demand access to specific treatments and pharmaceuticals [4]. Contrastingly, in countries like South Africa the judicialization of health care was spearheaded by rights-advocacy organizations who, acting collectively, went to Court in order to demand structural judicial remedies that challenged the global and local arrangements of intellectual property rights limiting access to HIV/AIDS drugs [5]. It is likely, as some authors suggest, that one of the variables that may explain why the individualized, case-by-case judicialization of health care in South Africa has been limited is the fact that in *common law* countries the weight of judicial precedent is much higher than in civil law countries like Brazil and Colombia [6].

Secondly, the judicialization of health care in Latin America is highly *pharmaceuticalized*, since most public expenditure related to health rights litigation is invested in paying for costly medications [7]. In Brazil, for example, the costs of health rights litigation are concentrated on a group of expensive biotech drugs for the treatment of chronical medical conditions like cancer, arthritis and rare diseases [8]. Similarly, a study found that most rulings handed down by Costa Rica’s Supreme Court provided costly, experimental or low-priority pharmaceuticals with only negligible health effects for plaintiffs [9]. In Colombia, the pharmaceuticalization of health care litigation has been a recurring issue in the literature and in the public debate. For instance, in 2008 the Colombian government invested US$156 million—approximately 22% of the public pharmaceutical expenditure—in paying health insurance companies for only seven types of expensive biotech drugs demanded, among others, by litigants [10]. A different, yet related instance of the pharmaceuticalization of health rights litigation is the financial support offered by Big Pharma companies to patients’ organizations that provide, in countries like Colombia, Brazil and Costa Rica, pro bono litigation support to patients living with cancer, HIV, renal failure and rare diseases, among other medical conditions [11].

In the past years, the optimism of the early socioeconomic rights literature, which celebrated the pioneering judicial decisions that enforced the right to health as a basic social right [12], has been replaced by a more precautionary approach that is attentive to the unintended and unwanted effects of the increasing litigation of health rights in Latin America. According to this literature, the judicialization of health care in Latin America may also be characterized by its lack of transformative potential. More concretely, a growing number of studies argue that the judicialization of health care exploits—financially— the health system but is incapable of transforming it [13]. Other scholars suggest that the judicialization of health care in Latin America is not transformative since it follows a *downstream* approach to litigation incapable of addressing the institutional, regulatory, environmental, and social determinants of health that lie *upstream* [14].

Among the most serious charges waged against the judicialization of health care in Latin America is that it fosters inequity in the allocation of limited health resources. In general terms, equity in health can be defined as “the absence of systematic [and potentially remediable] differences in one or more aspects of health status across socially, demographically, or geographically defined populations and subgroups” [15]. In this sense, health inequity refers to differences in access to health care that result from institutional arrangements that are potentially avoidable or remediable [16]. Therefore, not all inequalities can be considered inequitable. Only those health inequalities that are avoidable, unnecessary or remediable can be deemed as inequitable.

Most studies consider that equity in health services can be achieved by giving equal or *horizontal* treatment to equivalent needs [17]. However, the predominant notion of *horizontal equity*, which commends the equal treatment of equals, tends to overlook *vertical equity*, which suggests that in many cases policymakers must offer unequal or enhanced health services to those who have greater needs [18].

How to offer fair or equitable treatment to different health needs remains a nagging question in the literature. According to Norman Daniels’ *accountability for reasonableness* approach, in order to meet health needs fairly we have to rely on procedural justice. In other words, policymakers need to fall back on a process that is (a) transparent or public; (b) based on relevant reasons; (c) revisable through an appeals procedure; (d) enforceable [19].

When it comes to the literature on the judicialization of health care, despite the fact that there is cross-country evidence suggesting that the existence of a constitutional right to health contributes significantly to the wellbeing of citizens [20], a growing number of authors consider that the judicialization of health care is at odds with equity. As Biehl et al. argue, in the extant academic literature, but also in the journalistic portrayals of the judicialization of health care in countries like Brazil and Colombia, there is a prevailing narrative according to which judicialization is driven by urban elites and private interests and is used to access high-cost drugs that are not part of government formularies. It is reported that the people who file lawsuits are well-off litigants who exploit the expansiveness of the country’s constitutional right to health [21].

For instance, a study points out that in Colombia most health rights plaintiffs are not poor patients, but middle or upper-middle class individuals who, thanks to litigation, obtain access to expensive medical treatments [22]. Authors like Ferraz argue that in Brazil health rights litigation “harms the poor” since it has been hijacked by affluent free riders that use litigation to obtain high-cost medical treatments from the underfinanced public health system [23]. Based on that type of findings, Ferraz argues that in countries like Brazil health rights litigation is regressive because it skews limited health resources away from the poorest citizens and in favor of the more affluent [24].

But although the scholarship on the impact of health litigation on equity is growing, there are no studies that collect the available evidence and analyze the methodological soundness and significance of the literature’s findings. This paper seeks to provide such assessment by answering the following question: what type of evidence and methodological designs have been deployed to evaluate the impact of the judicialization of health on equity?

We focus on the leading developments in the extant literature to provide a framework to assess, based on a scoping review of the literature, the main characteristics of the studies that seek to understand whether the growing judicialization of health care in Latin America is conducive to more or less equitable health systems.

The findings of this paper can contribute not only to the specialized scholarship, but also to public policy and judicial reform discussions. On the one hand, if it is true that the judicialization of health care “harms the poor”, then the theoretical assumptions regarding the right to health’s redistributive potential [25] could be called into question.

On the other hand, if there is enough evidence supporting the claim that the judicialization of health care fosters inequity, governments, policy makers and legislators could trigger a wave of constitutional and legislative reforms aimed at curtailing the right to health and rights-based judicial mechanisms such as Colombia’s *Tutela*, Costa Rica’s *Amparo*, and Brazil’s *Mandado de Segurança*. This is already happening in Brazil, where local governments, pressed by the mounting financial pressure exerted by right to health litigation, have argued before the Federal Supreme Court (*Supremo Tribunal Federal* or STF) that health rights litigation benefits a few patients, but harms the health system as a whole with unreasonable demands for medical treatments and pharmaceuticals [26]. Taking into account those arguments, on April 2018 the STF decided that Brazil’s health system is only obliged to provide to litigants medications excluded from the basic health plan— *Sistema Único de Saúde* or SUS—under three conditions: that the medication is approved by ANVISA, the government’s food and drugs’ agency; that the litigant demonstrates that she is unable to pay for the medication; that the prescribing doctor states that medications included in the SUS plan are ineffective to treat the litigant, and that an “excluded” drug is thus needed [27].

However, if the evidence suggesting that the judicialization of health care is still inconclusive, as this paper finds, then a more precautionary approach to judicial reforms that may curtail health rights litigation is necessary.

## Methodology

In this paper we used a scoping study methodology, as described by Arksey and O’Malley [28] and by Levac et al. [29] In order to reduce the universe of results, we specifically searched for articles that addressed the effects of the judicialization of health care on equity in Latin American countries, independently of the study’s methodological design (theoretical, descriptive, qualitative, quantitative, mixed methods, etc.). Our search comprised journal articles, books and edited volumes. In the case of edited volumes, we treated book chapters as independent studies. For journal articles we searched Google Scholar, PubMed, Scopus, and Scielo databases. We also went through the studies’ bibliographic references, and hand-searched key journals and authors.

**Fig. 1:**
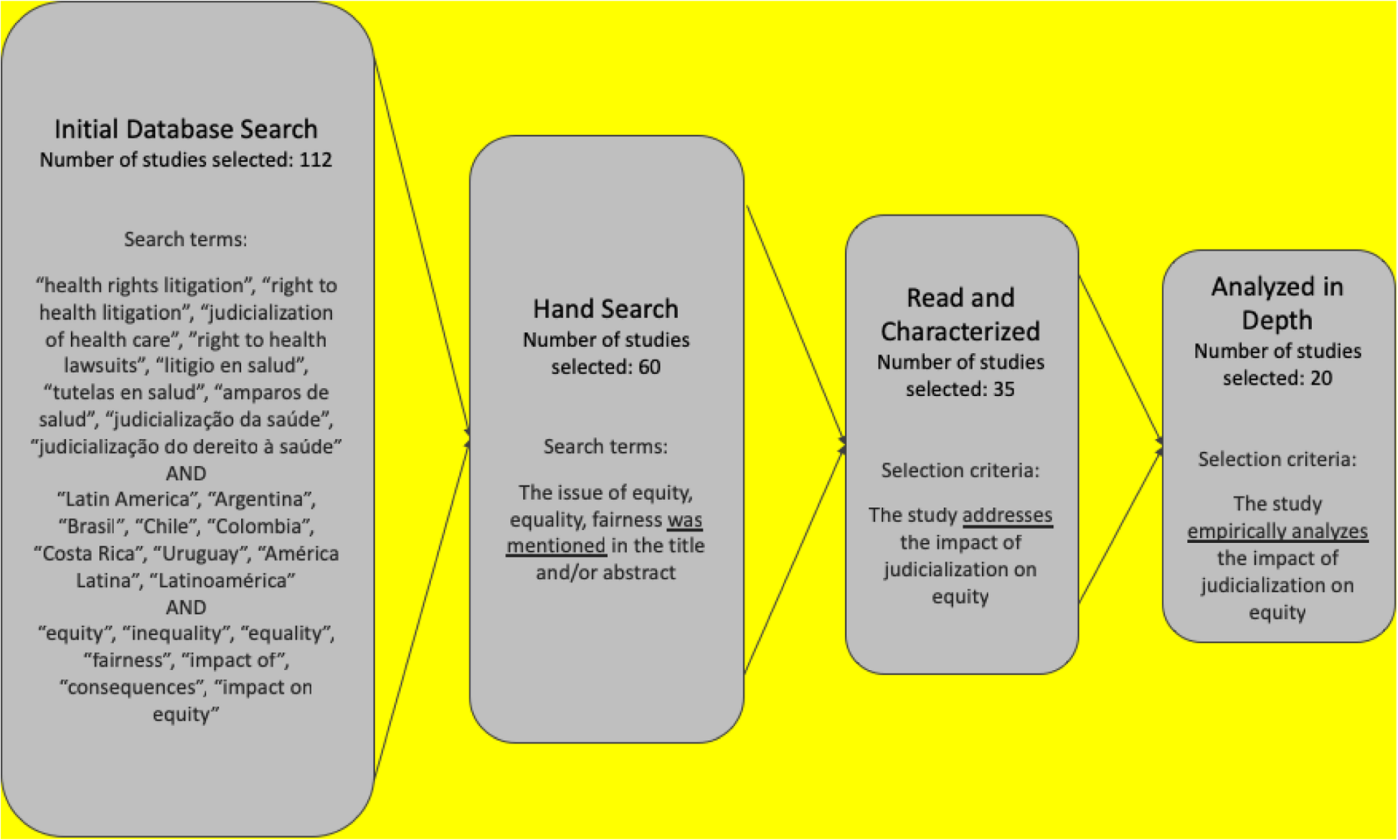
Studies’ selection strategy

Searches were conducted between February and April 2018. The initial search was circumscribed to studies written after 1990, when the pioneering judicial rulings on the right to health were handed down by higher Courts in Brazil, Colombia and Costa Rica. We combined search words such as “health rights litigation”, “judicialization of health care”, “right-to-health lawsuits”, “equity”, “fairness”, “impact”, and “consequences”, always in combination with “Latin America”, “Colombia”, “Brazil”, “Costa Rica”, “Argentina”, “right to health”, “amparo”, “tutela”, etc. More importantly, we included studies that used the word “equity”, “equality”, and “fairness”, irrespective of their different meanings in the literature (see Fig. 1 for all the search terms used) [30].

Of the initial 112 articles identified in the first search, 60 were selected based on the fact that they mentioned the issue of equity in their title and/or abstract. After reading the full text of those 60 studies, we found that only 35 actually addressed the equity consequences of the judicialization of health care (see Additional file 1: Annex 1 for a full list of these studies). In other words, in our initial sample of 112 pieces, only 35 studies dealt directly or indirectly with the question: is the judicialization of health care bad or good for equity? We then proceeded to characterize the 35 resulting articles using a set of more than 20 variables. The chosen variables seek to provide an overview of the general characteristics of these studies, what cases they choose, whether they are comparative, historic, interdisciplinary, and what type of methodologies, variables and data they use (see Table 1). Although not all variables analyzed here are directly related with the question of the impact of health care judicialization on equity, they help us understand who is concerned with this issue and where, why and how have the studies about it been conducted.

**Table 1:** Relevant variables to map the field of health care judicialization and equity in Latin America

Variables		Description		Values
Type of study	Title	Title of the study		Text
	N of Authors	Whether the study is authored by a single author or by multiple authors		Number
	Type of Publication	Whether it is a journal article, an entire book or a chapter in an edited volume		1 Article; 2 Book; 3 Edited Volume; 4 Policy Report
	Journal or Book title	Title of the publication		1 Public health; 2 Law; 3 Other
	Year	When it was published		Number
	Language	Language in which the study is written		Text
	Interdisciplinary	Whether authors belong to one or more disciplines		1 interdisciplinary; 0 disciplinary
		What disciplines the author(s) belong to?		Text
Cases of judicialization	Country	The country or countries the study refers to		Text
	City/Region	Whether the study focuses on a particular city or region within any given country		Text
	Empirical Equity Impact Assesment	Whether the study provides empirical data and analysis to assess impact on equity		1 provides empirical data and analysis; 0 descriptive with no empirical analysis
	Comparative	Whether it compares several countries or regional cases		1 yes; 0 no
	Dynamic perspective	Whether it tracks changes over time —in litigation patterns, type of litigants, etc.		1 yes; 0 no
	Type of Court	Whether the analysis is conducted at lower courts—state courts, city courts, municipal courts— or at the highest court in the land		1 Highest court; 2 Lower courts; 3 both
	Entitlements	Whether the study refers only to medicines or also to other treatments or procedures beyond pharmaceuticals.		1 Only medicines; 0 other medical treatments as well as medicines
Study design	Methods	Whether the study uses quantitative modeling, descriptive statistics, qualitative or mixed methods		1 Quantitative (models); 2 Quantitative (descriptive statistics); 3 Qualitative; 4 Mixed
		Whether the piece has an independent methods section		1 yes; 0 no
	Variables	Whether the following set of variables are analyzed:	Litigants’ demographics	1 yes; 0 no
			Type of legal representation	1 yes; 0 no
			Type of claims	1 yes; 0 no
			Prices or costs of litigation	1 yes; 0 no
			Other	Text
	Dataset	Whether it works with a data set		1 yes; 0 no
		Where does the data set come from		1 Totally constructed by the author(s); 2 Provided by the courts/government/litigants but heavily processed by the author(s); 3 Provided by a third party and taken as is
		How many observations does the data set have		Number
	Effect on equity	Whether the paper suggests that there is a positive, negative, or ambiguous effect of health litigation on equity		1 Positive; 2 Negative; 3 Ambiguous

We then investigated how many of the 35 studies conducted original empirical work. More concretely, we asked the following question: how many of the studies from our sample collected their own qualitative and/or quantitative data, totally or partially? We only included studies that conducted ethnographic work, extensive interviewing of stakeholders—litigants, judges, policymakers, NGOs, etc.—, applied surveys, or constructed robust databases based on content analysis of lawsuits, judicial rulings, courts’ files, etc. Based on the rationale that original empirical research is grounded on independent data gathering, we decided to exclude studies that declared themselves to be empirical, but only commented or synthetized data gathered by a third party—the government, other researchers, etc.

We found that 20 (57%) of the initial 35 studies constituted original empirical research (Fig. 1). We then proceeded to analyze this set of empirical studies. More concretely, we analyzed the type of equity approach the studies take, as well as quality variables that touched upon methodological and data collection strategies (see Table 2). The equity approach refers to whether the study explicitly or implicitly understands that citizens should receive equal treatment for equivalent needs (horizontal) or that preferential treatment should be given to those with greater health needs (vertical). We also assessed whether studies are concerned in anyway with procedural justice when allocating health care resources. The studies’ quality was measured using two sets of variables: (i) methodological design; and (ii) data collection and analysis’ techniques. The first set of variables evaluates whether there is a clear methodology, whether there is a fit between the question posed by the study and the methodology used to answer the question, how generalizable are the findings, and whether there are other questions that may be missing from the study given the methodological design. The second set of variables assesses how reliable and representative is the study’s data, whether the data is available or open for researchers and whether the analysis used in the paper can be replicated or not.

**Table 2:** Quality variables for selected empirical studies that assess the impact of health care judicialization on equity

Variables		Objective
Equity	Equity approach	Whether the study takes a horizontal or a vertical approach to equity
	Procedural justice	Whether it incorporates procedural justice concerns
Quality	Fit	How good is the fit between the research question and the methodological design
	Generalizability	Whether the findings are generalizable to other cases
	Data reliability	Whether the data used is reliable
	Data representativity	Whether the data used is representative of the case
	Replicability	Whether the study can be replicated (i.e. it has a clear methodological strategy and the data is available)
	Intercoder reliability test	Whether it has an IRT protocol or not
Type of litigant indicators	Location	Whether it incorporates and elaborates on the litigants’ place of residence
	Litigants’ relationship with health system	Public, private, contributory, subsidized, etc.
	Individual demographics	Age, education, income, etc.
	Intermediaries	Whether the litigant was represented by Attorney, patients’ organization, NGO, public attorney, physician
Type of claim indicators	Health benefits package (HBP)	Whether the claim is included in the HBP or not
	Prices	How much the claim costs
	Cost-effectiveness	Whether there is scientific evidence of the effectiveness of the treatment and/or whether it is cost-effective
Policy impacts	New policies	The creation of new public policies
	Effectiveness of current policies	The improvement of current policies
Symbolic impacts	Participation, deliberation, legitimacy, reframing	Whether judicialization of health care empowers, democratizes, etc.

## Results

In this section we present the most relevant results of our scoping exercise. We discuss the outcomes of our review of the initial set of 35 studies, including the 20 studies that we consider to be original empirical research. These findings allowed us to map out the general trends in the literature that addresses the impact of the judicialization of health care on equity (see Table 3 for a summary of the findings). Additionally, our results would allow us to assess the equity approach, and the methodological quality, scope, reliability, representability, and generalizability of the 20 empirical studies.

**Table 3:** General trends in the field of health care judicialization and equity in Latin America

Variables		Values
Type of study	N of Authors	Single author: 7 (20%)
		Two authors: 14 (40%)
		More than 2 authors: 14 (40%)
	Type of Publication	Article: 27 (77%)
		Chapter in an Edited Volume: 7 (20%)
		Policy Report: 1 (3%)
	Journal or Book title	Public health journals: 16 (46%)
		Law and public policy journals: 11 (31%)
		Other: 8 (23%)
	Year	2006–2010: 6 (17%)
		2011–2014: 23 (66%)
		2014–2018: 6 (17%)
	Language	English: 22 (63%)
		Spanish: 7 (20%)
		Portuguese: 6 (17%)
	Interdisciplinary	Interdisciplinary: 10 (28%)
		Disciplinary: 25 (72%)
Cases of judicialization	Country	Only Brazil: 19 (54%)
		Only Colombia: 8 (23%)
		Only Argentina: 3 (9%)
		Other single or multiple countries: 5 (14%)
	Empirical Equity Impact Assesment	Provides empirical data and analysis: 24 (69%)
		Theoretical or descriptive: 11 (31%)
	Comparative	Comparative: 4 (11%)
		Single case: 31 (89%)
	Dynamic perspective	Patterns over time: 6 (17%)
		Static: 29 (83%)
	Type of Court	Highest court: 9 (26%)
		Lower courts: 20 (57%)
		Both or N/A: 6 (17%)
	Entitlements	Only medicines: 14 (40%)
		Other medical treatments: 21 (60%)
Study design	Methods	Quantitative (models): 0 (0%)
		Descriptive statistics: 26 (74%)
		Qualitative: 9 (26%)
		Methods section: 19 (54%)
		No methods section: 16 (46%)
	Variables	Litigants’ demographics: 20 (57%)
		Type of legal representation: 19 (54%)
		Type of claims: 26 (75%)
		Prices or costs of litigation: 11 (31%)
	Dataset	Totally constructed by the author(s): 20 (57%)
		Other: 15 (43%)
	Effect on equity	Positive: 7 (20%)
		Negative: 17 (49%)
		Ambiguous: 11 (31%)

### General trends in the literature

#### Type of studies

Of the 35 studies reviewed, the majority (77%, 27) were journal articles. The remaining studies were book chapters and policy papers. More than half (59.2% 16) of the articles were published in public health journals, while 40.8% [11] appeared in law and public policy journals. 63% [22] of all studies were written in English, whereas 17% [6] were written in Portuguese and 20% [7] in Spanish.

**Fig. 2:**
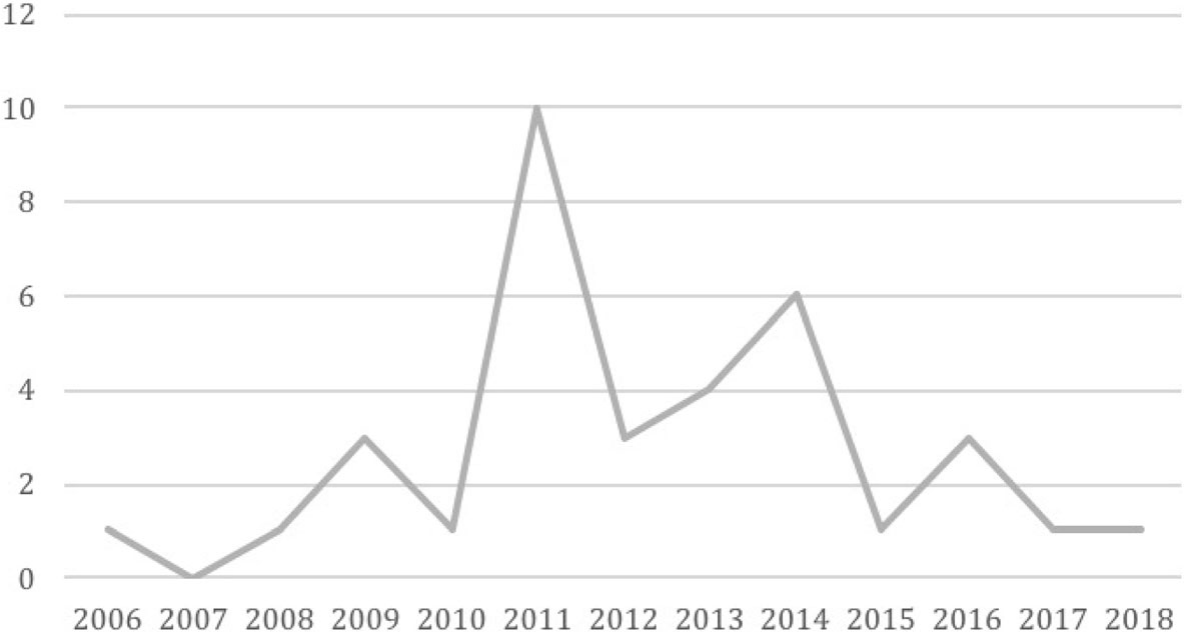
Articles per year

The majority of articles (65%, 23) were published between 2011 and 2014 (see Fig. 2). We also found that the type of research varied over time. While pioneer studies focused on all types of health-related claims and demands raised by litigants, recent studies tend to focus on treatments for specific diseases and health conditions—such as diabetes, cancer or rare diseases. After 2009, there was a marked interest in pharmaceuticals demanded by plaintiffs: 40% [14] of studies chose to analyze only pharmaceutical judicial claims and their impact on equity.

A comparatively small percentage of studies (28%, 10) were authored by an interdisciplinary team of scholars. A large percentage of the reviewed studies (47%, 16) were written by legal experts. Interestingly, economists and health economists are underrepresented in our sample: only 3 (8%) studies authored by interdisciplinary teams include economists.

#### Judicialization

**Fig. 3:**
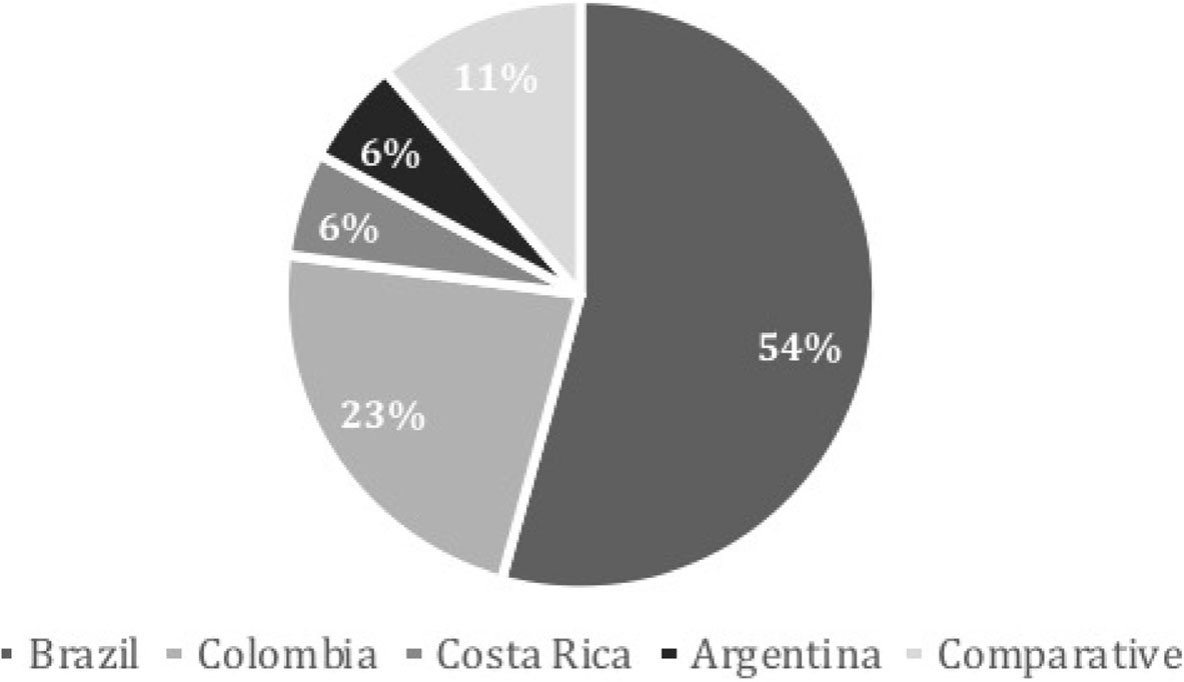
Studies by country

Only a small percentage of the studies adopt a comparative approach (11%, 4); the rest conduct case studies at the national and subnational level. 54% [19] of studies address the case of Brazil, which is by far the most analyzed country in the selected literature (see Fig. 3). It is interesting to note that an important part of the studies that address only the Brazilian case conduct research at the subnational level (44.4%, 15), especially for states, regions and cities such as São Paulo, Rio Grande do Sul, Rio de Janeiro, among others. The second most studied case is Colombia, with 23% [8] of all cases, followed by Costa Rica and Argentina, each with only 2 studies (11%). It is also interesting to note that all the studies that deal with the Colombian and Costa Rican cases ignore the subnational level and focus on the national level. Besides Brazil, only the Argentinian studies incorporate research conducted at the subnational level, especially for the case of the Buenos Aires city-region. This may be explained by the fact that Colombia and Costa Rica are centralized countries while Argentina and Brazil have federal political systems.

In the cases of Brazil and Argentina, data analysis was done at lower courts. In contrast, the data analysis in Colombia and Costa Rica took place at the highest court in the land, usually using the search engines provided by the judicial branch or governmental agencies like the ombudsman office or the public attorney’s office. Most studies that analyze the Colombian case tend to address the impact of structural judicial rulings that order the executive branch to reform the health system, such as the CCCs ruling T-760/08. In the case of Argentina, studies focus not only on individual litigation but also on collective and class action litigation (*amparo colectivo*).

In our database, only 6 (17%) studies look at changes in litigation patterns over time. It is relevant that 4 of those 6 studies address the Colombian case. By contrast, all the studies about the Brazilian case, except one [31], ignore possible changes in judicialization patterns over time.

#### Assessing impact on equity: databases, variables and indicators

All the studies analyzed in our scoping exercise use qualitative data or descriptive statistics with simple correlations. In our database there are no examples of quantitative studies that deploy econometric models, or any other kind of methods used to evaluate causation. Half of the studies (53%, 18) have a methods section that describes the methodological tools followed by authors.

Most of the studies we reviewed (56%, 20) construct databases in order to characterize judicialization and assess its impact on equity. Samples can vary markedly, going from 18 to 4.343 observations. While authors who study the judicialization of health care in Brazil tend to construct their own databases, papers and chapters devoted to other Latin American countries rely heavily on governmental/judicial branch datasets and search engines. For instance, most of the studies that deal with the Colombian case were conducted using governmental reports published either by the Ombudsman’s Office or by the Ministry of Health, which sometimes lead researchers to contradictory results.

One important conclusion of the scoping exercise is that most studies in our data base focus on the type of litigants and/or the type of health services and technologies demanded by plaintiffs. Very few studies empirically address the judges’ decision-making process [32] or the intermediaries involved in the legal representation of plaintiffs, such as attorneys or public defendants [33].

The most recurrent variables analyzed by the studies in our sample are the type of claims made by litigants, followed by the socioeconomic status of litigants and their type of legal representation. 75% [26] of studies look at what type of claims are made by plaintiffs –drugs, services, procedures, etc. 57% [20] of the studies measure litigants demographics in some way –place of residence, income, education, age, gender, etc.—and 54% [19] investigate the type of legal representation received by plaintiffs –public or private attorneys, patients’ organizations, etc. Furthermore, 31% [11] of the studies indagate about costs or prices of medications demanded by plaintiffs.

Finally, 49% [17] of the studies reported that the judicialization of health care has negative effects on equity, 31% [11] showed inconclusive results, and 20% [7] found a positive effect.

### Trends in the empirical literature

#### Equity approach

Even if none of the 20 empirical studies we analyzed in depth provides a systematic discussion of what equity means, their equity approach can be extracted from the type of research conducted and from the motivation and concluding statements. The main concern of these empirical studies is that the judicialization of health care, by securing individual needs, is in tension with the attainment of collective benefits. Some of the studies analyze this intervention by focusing on who are the individuals getting access to health care by means of judicialization, while others focus on the costs, financial and/or organizational, of the judicialization of health care. In both of these approaches, equity is understood as a matter of procedural justice by which judicialization interferes with the standing processes of allocation of scarce health care resources. This intervention is not necessarily negative, for 4 (20%) of the studies judicialization has positive effects and for 3 (15%) it has ambiguous effects on equity because it gives voice to citizens that would otherwise not have it, making the health care decision-making process more transparent and accountable.

Regarding horizontal or vertical equity, given that the judicialization of health care privileges litigants over non-litigants it negatively affects horizontal equity. However, the majority of the studies (60%, 12) take a vertical approach to equity, by discussing whether the judicialization of healthcare addresses the claims of the neediest individuals. For some of the studies need has mainly to do with socio-economic vulnerability, and thus they ask whether judicialization benefits the poorest. For other studies need has also to do with health needs, and thus they are concerned with whether judicialization benefits the sickest.

#### Quality of empirical evidence

We conducted an in-depth analysis of the 20 studies that carried out original empirical research. An important result to report is that all studies have limited generalizability, either because their sample is unrepresentative, or because despite having a representative sample they concentrate on a single region, medical condition, or type of pharmaceutical. All of the 20 studies use descriptive statistics to analyze their databases, which in a few cases is complemented with interviews or ethnographic observations. However, no single study makes its database available for other research teams, all of which reduces reliability and precludes replicability.

The Brazilian empirical literature outnumbers any other country in our sample. 75% [15] of the 20 empirical studies are about Brazil, 2 about Argentina, 1 about Costa Rica, 1 about Colombia and 1 is a comparative study including Brazil, Colombia, and Costa Rica. All of the studies use indicators such as litigants’ profiles and types of claims to evaluate whether the judicialization of health care is good or bad for equity. Only 2 studies look at policy changes as an indicator relevant for equity.

The 20 empirical studies from our sample use several indicators to characterize litigants and to evaluate whether health judicialization is contributing to inequity. They include direct demographic indicators such as income, place of residence, age, gender and education, and indirect indicators of social status such as the type of health services—public or private—habitually used by the claimant, or whether litigants hired a private lawyer or were represented by a public attorney.

Regarding the type of health services and technologies demanded by litigants, our empirical sample shows that most of the studies focus on the type of pharmaceuticals and treatments demanded by litigants. Additionally, most studies ask whether the pharmaceuticals and treatments demanded by plaintiffs are included in the health benefit packages, whether those treatments and pharmaceuticals are approved by a governmental regulatory agency or not, how much do they cost and whether they have cheaper alternatives in the market. There are 4 studies that assess whether pharmaceuticals and treatments are cost-effective and whether there is enough scientific evidence supporting their treatment’s effectiveness.

Studies that conclude that the judicialization of health care is bad for equity usually respond to two types of questions: Who litigates, and what is being litigated. The main argument regarding who litigates is that plaintiffs are well-off individuals who skew limited health resources away from more needy patients. On the other hand, the question of what is being litigated is usually answered by pointing out that courts oblige health systems to provide ineffective pharmaceuticals whose high costs dislodge the fair allocation of limited health resources, all of which contributes to the furthering of inequity.

Only 4 of the empirical studies that we reviewed consider how the judicialization of health care may have indirect effects, either symbolic or instrumental. Following Rodriguez and Rodriguez, indirect symbolic effects of judicial rulings refer to judicial opinions ability to reframe the way public opinion perceive a social phenomenon, for example health care as a basic right. Indirect instrumental effects refers to the formation of advocacy coalitions that can influence decision-making processes regarding the litigated issue [34]. This type of indirect effects is relevant in terms of equity because, as illustrated by Rodriguez and Rodriguez for the case of the rights of displaced population, changes in the perception of an issue or the advocacy around it can lead to gradual institutional or policy changes that better guarantee the right in question. For example, health litigation can influence the update of benefit packages, it can nudge the creation price controls for pharmaceuticals, or it can boost public debates about the fairness of the health system as whole. This type of indirect effects of the judicialization of health care are mentioned in the literature but not properly assessed by means of empirical research.

## Conclusion

Is the judicialization of health care bad for equity? According to this review we are far from reaching a consensus on this issue. Half of the studies analyzed here find that judicialization has a negative impact on equity, but the other half finds that evidence is inconclusive or that the judicialization of health care has a positive effect on equity.

Although the equity approaches vary across studies, there seems to be a consensus that judicialization interferes with the regular allocation of health care resources; whether this is a good or a bad thing is where the whole debate lies. However, when measuring the impacts of the judicialization of health care on equity studies privilege the vertical approach to equity focusing on whether litigation benefits those who need it the most. Only some studies try to assess the procedural impacts of the judicialization of health addressing the fiscal, organizational and opportunity costs associated with litigation.

Moreover, the evidence and the methodologies used by the extant literature to assess the impact of health care judicialization on equity are, in most cases, weak. Three main issues regarding evidence arise from the analysis provided here. Firstly, comprehensive and reliable data is a problematic issue. Secondly, the majority of the studies that collect their own data rely on limited samples that are sometimes not representative and mostly not generalizable. Thirdly, there are several indicators that remain unexplored by the current literature but that could shed light on the impact of judicialization on equity. For example, on the litigants’ side, whether there are repeated claims by the same litigants, like orphan diseases patient’s associations and networks, could mean greater resources and higher rates of success in courts for patients with those conditions than for patients with more prevalent diseases that litigate only once (the *repeat player* hypothesis [35]). This, in turn, can provide new insights on the equity effects, vertical, horizontal and procedural, of the judicialization of healthcare. On the claims’ side, we still lack reliable accounts about public expenditure on expensive and ineffective treatments vis-a-vis cost-effective treatments that were first litigated and then included in health benefit packages—thanks, in great part, to the pressure exerted by litigation on policymakers. For example, a recently published paper suggests that in the case of Brazil, the changes brought about by individual litigation have potential to contribute to efficiency and fairness in the health care system through the improvement of the Health Technology Assessment (HTA) decision-making processes and health care governance [36]. Finally, the literature has not explored whether there are significant changes in the content of lawsuits across time that may reflect the emergence of different health needs within a given population.

Regarding the methodological strategies we find several gaps in the extant literature. Firstly, only few studies conduct systematic comparative analysis of different cross-country or within-country cases. Secondly, methodological approaches are focused only on the demographics of litigants and the availability, price and cost-effectiveness of treatments claimed, but tend to disregard symbolic and public policy impacts. For example, there are no empirically grounded analysis of the impact of judicialization on public policies such as cost-containment regulation, transparency of the relationship between pharmaceutical companies, physicians and patients, or on public debates about the limits of public health expenditure that could improve the allocation of healthcare resources. If, as Borges suggests, health litigation leads to better public policies that improve efficiency, these types of effects have to be factored in before one could argue for or against the judicialization of healthcare. Thirdly, none of the studies reviewed here aim to establish causation between judicialization and health outcomes (for instance, did the litigant’s health improve after the lawsuit?) that could give us a clearer view about the impact of the judicialization of health care on equity. Lastly, interdisciplinarity is limited and there is little methodological innovation.

Moreover, the literature tends to be categorical and ideological when it touches upon the issue of equity and litigation. An emblematic example is the divide that exists in Brazil between a pro-litigation camp that attributes to judicialization a positive role in guaranteeing the equitable right to health, and an anti-litigation camp, which argues that judicialization deepens health inequalities.

In conclusion, the findings of this article suggest that we still lack conclusive evidence about the regressive effect of health rights litigation in Latin America. In order to prove or disprove that the judicialization of health care is a foe for equity we first need to overcome the methodological and research-design problems that we identified in the studies analyzed here. There is ample space for innovation both in variables and in methods. Issues like changes in regulation, state capacity, the behavior of litigants, prescribers and judges, and the economic interests of big-pharma, are variables that could prove useful if they are incorporated into a rigorous empirical literature capable of assessing the regressive or progressive effect of health rights’ litigation.

## Additional file

Additional file 1: **Annex 1.** Studies selected. (DOCX 28 kb)

## Abbreviations

ANVISA: Agência Nacional de Vigilância Sanitária (Brazilian Health Regulatory Agency)
CCC: Colombian Constitutional Court
HTA: Health Technology Assesment
STF: Supremo Tribunal Federal (Brazil’s Federal Supreme Court)
SUS: Sistema Único de Saúde (Brazil’s Basic Health Care System)
