# Supplementary material for: Is the judicialization of health care bad for equity? A scoping review
Source: Int J Equity Health. 2019 Jun 3;18:61. doi: 10.1186/s12939-019-0961-y (PMC6545687; doi:10.1186/s12939-019-0961-y)
Supplement: Supplementary file 1 — Annex 1. Studies selected. (DOCX 28 kb) [file 12939_2019_961_MOESM1_ESM.docx]

**Additional file 1: Annex 1. Studies selected**

| **N** | **In depth** | **Title** | **Year** | **Author(s)** | **Journal or Book name or Report series** | **Editors** | **Country** | **Effect on Equity** |
| --- | --- | --- | --- | --- | --- | --- | --- | --- |
| 1 | Yes | Distortions to national drug policy caused by lawsuits in Brazil | 2006 | Fabiola Sulpino Vieira and Paola Zucchi | Revista Saude Pública |  | Brazil | Negative |
| 2 | No | El derecho a la salud en los tribunales. Algunos efectos del activismo judicial sobre el sistema de salud en Argentina | 2008 | Víctor Abramovich and Laura Pautassi | Salud Colectiva |  | Argentina | Positive |
| 3 | No | The Right to Health in the courts of Brazil: Worsening Health Inequities? | 2009 | Octavio Luiz Motta Ferraz | Health and human rights |  | Brazil | Negative |
| 4 | No | How Do Courts Set Health Policy? The Case of the Colombian Constitutional Court | 2009 | Alicia Yamin and Oscar Parra | Policy Forum |  | Colombia | Ambiguos |
| 5 | Yes | "Judicialization" of public health policy for distribution of medicines | 2009 | Ana Luiza Chieffi and Rita Barradas Barata | Cadernos de Saude Pública |  | Brazil | Negative |
| 6 | No | Judicial Protection of the Right to Health in Colombia: From Social Demands to Individual Claims to Public Debates | 2010 | Alicia Yamin and Oscar Parra | Hastings International and Comparative Law Review |  | Colombia | Ambiguos |
| 7 | No | Assesing the impact of health rights litigation: a comparative analysis of Argentina, Brazil, Colombia, Costa Rica, India and South Africa | 2011 | Ottar Mastad, Lise Rakner, Octavio Ferraz | Litigating Health Rights: Can Courts Bring More Justice to Health? | Alicia Yamin and Siri Gloppen | Argentina, Brazil, Colombia, Costa Rica, India, South Africa | Ambiguos |
| 8 | Yes | Health inequalities, rights, and courts: the social impacts of the judicialization of health | 2011 | Octavio Luiz Motta Ferraz | Litigating Health Rights: Can Courts Bring More Justice to Health? | Alicia Yamin and Siri Gloppen | Brazil | Negative |
| 9 | No | Judicial protection of the right to health: and elusive promise? | 2011 | Alicia Yamin, Oscar Parra-Vera, Camila Gianella | Litigating Health Rights: Can Courts Bring More Justice to Health? | Alicia Yamin and Siri Gloppen | Colombia | Positive |
| 10 | Yes | Courts and the right to health: achieving fairness despite "routinization" in individual coverage cases | 2011 | Paola Bergallo | Litigating Health Rights: Can Courts Bring More Justice to Health? | Alicia Yamin and Siri Gloppen | Argentina | Positive |
| 11 | No | Beyond the Courtroom: The Impact of Judicial Activism on Socioeconomic Rights in Latin America | 2011 | César Rodríguez Garavito | Texas Law review |  | Colombia | Ambiguos |
| 12 | No | Harming the Poor Through Social Rights Litigation: Lessons from Brazil | 2011 | Octavio Luiz Motta Ferraz | Texas Law review |  | Brazil | Negative |
| 13 | Yes | Claiming the Right to Health in Brazilian Courts: The Exclusion of the already Excluded? | 2011 | Virgilio Afonso da Silva and Fernanda Vargas Terrazas | Law and Social Inquiry |  | Brazil | Negative |
| 14 | Yes | Judicialization of access to medicines in Minas Gerais state, Southeastern Brazil | 2011 | Marina Amaral de Ávila MachadoI,II Francisco de Assis AcurcioII Cristina Mariano Ruas BrandãoIII,IV Daniel Resende FaleirosV Augusto Afonso Guerra JrV Mariângela Leal CherchigliaIV Eli Iola Gurgel AndradeIV | Revista Saude Pública |  | Brazil | Negative |
| 15 | Yes | Litigating for medicines: how can we access impact on health outcomes | 2011 | Ole Frithjof Norheim and Siri Gloppen | Litigating Health Rights: Can Courts Bring More Justice to Health? | Alicia Yamin and Siri Gloppen | Brazil, Colombia, Costa Rica | Negative |
| 16 | Yes | Costos de los servicios de salud tutelados y del proceso legal de las tutelas en Medellín, 2009 | 2011 | Emmanuel Nieto y Alejandro Arango | Revista Facultad Nacional de Salud Pública |  | Colombia | Negative |
| 17 | No | La judicialización de la salud: síntomas, diagnóstico y prescripciones | 2012 | César Rodríguez Garavito | La Salud en Colombia Logros, retos y recomendaciones | Óscar Bernal y Catalida Gutiérrez | Colombia | Ambiguos |
| 18 | No | Universal health coverage and litigation in Latin America | 2012 | Leonardo Cubillos, Maria-Luisa Escobar, Sebastian Pavlovic, Roberto Iunes | Journal of Health Organization and Management |  | Latin America | Negative |
| 19 | Yes | Doctors, lawyers and pharmaceutical industry on health lawsuits in Minas Gerais, Southeastern Brazil | 2012 | Orozimbo Henriques Campos NetoI Francisco de Assis AcurcioII Marina Amaral de Ávila MachadoI Felipe FerréIII Fernanda Loureiro Vasconcelos BarbosaIV Mariângela Leal CherchigliaV Eli Iola Gurgel AndradeV | Revista Saude Pública |  | Brazil | Negative |
| 20 | No | La judicialización de la salud en Colombia: el desafío de lograr los consensos hacia mayor equidad y cobertura universal | 2013 | Juanita Durán y Rodrigo Uprimny | Pactos sociales para una protección social más inclusiva Experiencias, obstáculos y posibilidades en América Latina y Europa | Martín Hopenhayn, Carlos Maldonado Varela, Rodrígo Martínez, María Nieves Rico y Ana Sojo | Colombia | Ambiguos |
| 21 | No | The Debatable Role of Courts in Brazil's Health Care System: Does Litigation Harm or Help? | 2013 | Mariana Mota Prado | Journal of Law, Medicine and Ethics |  | Brazil | Ambiguos |
| 22 | Yes | REACHING OUT TO THE NEEDY? ACCESS TO JUSTICE AND PUBLIC ATTORNEYS' ROLE IN RIGHTTO HEALTH LITIGATION IN THE CITY OF SAO PAULO | 2013 | Daniel Liang Wang and Octavio Luiz Motta Ferraz | Sur |  | Brazil | Negative |
| 23 | Yes | The thesis of judicialization of health care by the elites: medication for mucopolysaccharidosis | 2013 | Marcelo Medeiros 1 Debora Diniz 2 Ida Vanessa Doederlein Schwartz | Ciencia & Saude Colectiva |  | Brazil | Ambiguous |
| 24 | No | Right to health, essential medicines, and lawsuits for access to medicines: A scoping study | 2014 | Claudia Marcela Vargas-Pelaez, Marina Raijche Mattozo Rover, Silvana Nair Leite, Francisco Rossi Buenaventura, Mareni Rocha Farias | Social Science & Medicine |  | World | Negative |
| 25 | No | Equidad y protección judicial del derecho a la salud en Colombia | 2014 | Rodrigo Uprimny y Juanita Durán | Políticas Sociales | CEPAL | Colombia | Ambiguos |
| 26 | Yes | Health Rights Litigation and Access to Medicines: Priority Classification of Successful Cases from Costa Rica’s Constitutional Chamber of the Supreme Court | 2014 | Ole Frithjof Norheim and Bruce M. Wilson | Health and human rights |  | Costa Rica | Negative |
| 27 | Yes | Os impactos da judicialização da saúde no município de São Paulo: gasto público e organização federativa | 2014 | Daniel Wei L. Wang, Natália Pires de Vasconcelos, Vanessa Elias de Oliveira, Fernanda Vargas Terrazas | Revista Admin Pública |  | Brazil | Negative |
| 28 | No | Itinerário dos usuários de medicamentos via judicial no estado do Amazonas, Brasil | 2014 | Marselle Nobre de Carvalho(a) Silvana Nair Leite | Comunicacao saude educacao |  | Brazil | Positive |
| 29 | Yes | The judicialization of health in the Federal District of Brazil | 2014 | Debora Diniz 1 Teresa Robichez de Carvalho Machado 2 Janaina Penalva | Ciencia & Saude Colectiva |  | Brazil | Positive |
| 30 | Yes | Access to treatment for phenylketonuria by judicial means in Rio Grande do Sul, Brazil | 2015 | Luciano Mangueira Trevisan 1 Tatiele Nalin 1 Tassia Tonon 1 Lauren Monteiro Veiga 2 Paula Vargas 3 Bárbara Corrêa Krug 4 Paulo Gilberto Cogo Leivas 2 Ida Vanessa Doederlein Schwartz | Ciencia & Saude Colectiva |  | Brazil | Positive |
| 31 | Yes | The Judicialization of Health and the Quest for State Accountability: Evidence from 1,262 Lawsuits for Access to Medicines in Southern Brazil | 2016 | João Biehl, Mariana P. Socal, and Joseph J. Amon | Health and human rights |  | Brazil | Positive |
| 32 | Yes | Judicialization of the right to health in the Brazilian Northeastern region: dimensions and challenges | 2016 | Carlos Francisco Oliveira Nunes and Alberto Novaes Ramos Júnior | Cadernos de Saude Coletiva |  | Brazil | Negative |
| 33 | Yes | Litigation and the right to health in Argentina | 2016 | Verónica Gotlieb 1 Natalia Yavich 1 Ernesto Báscolo | Cadernos de Saude Pública |  | Argentina | Ambiguous |
| 34 | Yes | What is the cost of brand name prescriptions in the judicialization of access to medicines? | 2017 | Luís Fernando Nunes Alves Paim, Carine Raquel Batt, Gabriela Saccani, Irene Clemes Küllkamp Guerreiro | Cadernos de Saude Coletiva |  | Brazil | Negative |
| 35 | Yes | HEALTH JUDICIALIZATION: ACCESS TO TREATMENT FOR USERS WITH DIABETES MELLITUS | 2018 | Ellen Cristina Barbosa dos Santos, Carla Regina de Souza Teixeira, Maria Lúcia Zanetti, Plínio Tadeu Istilli, Lúcia Helena Terenciani Rodrigues Pereira, Maria Teresa da Costa Gonçalves Torquato | Texto Contexto Enfermeria |  | Brazil | Ambiguous |
